# Health status of children and young persons with congenital adrenal hyperplasia in the UK (CAH-UK): a cross-sectional multi-centre study

**DOI:** 10.1530/EJE-21-1109

**Published:** 2022-08-24

**Authors:** Irina Bacila, Neil Richard Lawrence, Sundus Mahdi, Sabah Alvi, Timothy D Cheetham, Elizabeth Crowne, Urmi Das, Mehul Tulsidas Dattani, Justin H Davies, Evelien Gevers, Ruth E Krone, Andreas Kyriakou, Leena Patel, Tabitha Randell, Fiona J Ryan, Brian Keevil, S Faisal Ahmed, Nils P Krone

**Affiliations:** 1 Department of Oncology and Metabolism, University of Sheffield, Sheffield, UK; 2 Leeds General Infirmary, Leeds, UK; 3 Great North Children’s Hospital, University of Newcastle, Newcastle, UK; 4 Bristol Royal Hospital for Children, University Hospitals Bristol Foundation Trust, Bristol, UK; 5 Alder Hey Children’s Hospital, Liverpool, UK; 6 Great Ormond Street Hospital, London, UK; 7 University Hospital Southampton, Southampton, UK; 8 Southampton, United Kingdom University of UK; 9 Centre for Endocrinology, William Harvey Research Institute, Queen Mary University London, London and Barts Health NHS Trust – The Royal London Hospital, London, UK; 10 Birmingham Women’s & Children’s Hospital, Birmingham, UK; 11 Developmental Endocrinology Research Group, University of Glasgow, Glasgow, UK; 12 Paediatric Endocrine Service, Royal Manchester Children’s Hospital, Manchester University NHS Foundation Trust, Manchester, UK; 13 Nottingham Children’s Hospital, Nottingham, UK; 14 Oxford Children’s Hospital, Oxford University Hospitals NHS Foundation Trust, Oxford, UK; 15 Department of Biochemistry, Manchester University NHS Foundation Trust, Manchester, UK

## Abstract

**Objective:**

There is limited knowledge on the onset of comorbidities in congenital adrenal hyperplasia (CAH) during childhood. We aimed to establish the health status of children with CAH in the UK.

**Design and methods:**

This cross-sectional multicentre study involved 14 tertiary endocrine UK units, recruiting 101 patients aged 8–18 years with classic 21-hydroxylase deficiency and 83 controls. We analysed demographic, clinical and metabolic data, as well as psychological questionnaires (Strengths and Difficulties (SDQ), Paediatric Quality of Life (PedsQL)).

**Results:**

Patient height SDS in relation to mid-parental height decreased with age, indicating the discrepancy between height achieved and genetic potential height. Bone age was advanced in 40.5% patients, with a mean difference from the chronological age of 1.8 (±2.3) years. Patients were more frequently overweight (27%) or obese (22%) compared to controls (10.8% and 10.8%, respectively, *P* < 0.001). No consistent relationship between glucocorticoid dose and anthropometric measurements or hormonal biomarkers was detected. A small number of patients had raised total cholesterol (3.0%), low HDL (3.0%), raised LDL (7.0%) and triglycerides (5.0%). SDQ scores were within the ‘high’ and ‘very high’ categories of concern for 16.3% of patients. ‘School functioning’ was the lowest PedsQL scoring dimension with a median (interquartile range) of 70 (55–80), followed by ‘emotional functioning’ with a median of 75 (65–85).

**Conclusions:**

Our results show an increased prevalence of problems with growth and weight gain in CAH children and suggest reduced quality of life. This highlights the urgent need to optimise management and monitoring strategies to improve long-term health outcomes.

## Introduction

Congenital adrenal hyperplasia (CAH) represents a group of autosomal recessive conditions with altered adrenal steroid synthesis. The classic form of 21-hydroxylase deficiency (21OHD) accounts for most cases affecting approximately 1:15 000 individuals (1, 2). It presents with glucocorticoid (GC) deficiency and androgen excess, as well as clinically apparent mineralocorticoid deficiency in 75% of cases (1, 2, 3). Patients require lifelong hormone replacement, including GCs at relatively high doses to replace cortisol and normalise androgen excess (4). The management of CAH is challenging, as currently used synthetic GCs cannot mimic the physiological cortisol rhythm, especially given the broad inter-individual pattern of GC metabolism. In the absence of an optimal treatment strategy, there is variability in management among different countries and centres (5). Furthermore, an increased rate of comorbidities has been described in adults with CAH, including cardiovascular and metabolic disease and reduced fertility (1, 6, 7, 8). As the evidence regarding the health status of children and young persons with CAH is limited, it is difficult to establish the optimal timing for targeting secondary prevention strategies. Previous single-centre studies provided limited information on the topic due to the relatively small number of participants (9, 10). A clinical consortium formed of investigators from 14 tertiary UK centres was established in February 2015 (CAH-UK) to address vital clinical questions, working in collaboration with medical professionals, the UK-CAH support group (www.livingwithcah.com), patients and parents. This study establishes for the first time the onset of CAH-associated comorbidities and impaired quality-of-life (QoL) in relation to markers of disease control and corticosteroid replacement in children with CAH.

## Methods

This cross-sectional, multicentre study involving 14 tertiary UK centres (Supplementary Table 1, see section on supplementary materials given at the end of this article) recruited 107 patients with 21OHD and 83 controls. The study was approved by the Yorkshire and Humber Research Ethics Committee (Clinical Trials Registration Number: SCH/15/088).

Recruitment took place between September 2016 and December 2018, according to the inclusion criteria (Supplementary Table 2). Patients were recruited locally using information leaflets and advertisements in the UK-CAH support group newsletter. Healthy age- and sex-matched controls were recruited by advertisements placed within associated universities, NHS trusts and GP surgeries.

Patients attended visits at their respective centres between 08:00 and 09:00 h, in fasted state, having taken their usual morning replacement medication (Supplementary File 1). Blood samples were collected, and then patients underwent a physical examination including measurement of height, weight, waist and hip circumference, four-limb blood pressure and assessment of pubertal development. A comprehensive medical history was taken from the notes, patients and parents, including initial presentation, history of adrenal crisis, genital surgery, current medication and the most recent bone age. Patients and parents were also asked to complete QoL questionnaires. Controls underwent similar visits, with the following distinctions: self-rated pubertal stage and no QoL questionnaires.

### QoL questionnaires

QoL questionnaires consisted of patient and parent Paediatric Quality of Life (PedsQL™Generic Core Scale) and Strengths and Difficulties Questionnaires (SDQ) (https://www.sdqinfo.org) (11), administered in paper format, results being analysed according to the respective established protocols.

### Local sample analysis

Biochemical profiles (electrolytes, urea, lipids, glucose and plasma renin/renin activity) were analysed in accredited local NHS laboratories and interpreted based on local normal ranges.

### Centralised sample analysis

Blood samples were collected locally, and then sent to the collaborating laboratories for analysis. The measurement of blood steroid hormones (17-hydroxyprogesterone and androstenedione) was conducted by liquid chromatography-tandem mass spectrometry in the Biochemistry Department, University Hospital of South Manchester. Results were interpreted in accordance with previously recommended ranges (12, 13). Blood insulin measurement by ELISA was conducted centralised at the Clinical Chemistry Department, Sheffield Teaching Hospital. Insulin resistance was assessed based on the homeostatic model of insulin resistance (HOMA-IR) using the formula: fasting insulin (µU/L) × fasting glucose (nmol/L)/22.5, with the cut-off of 1.68 for normal-weight and 3.42 for overweight individuals (14).

Body surface area and mid-parental height were calculated using the Mosteller and Tanner’s formulas, respectively. Participants were classified according to BMI as defined by the World Health Organization (overweight > +1 s.d., obesity > +2 s.d.) (15). Delta height SDS was calculated as the difference between patient height SDS and mid-parental height SDS. High blood pressure was defined by a systolic and/or diastolic pressure above the 95th percentile for age, sex and height in all four-limb measurements (16).

### Statistical analysis

Descriptive statistics were used to express demographic characteristics and treatment doses. Chi-square test, ANOVA, Mann–Whitney U test and Fisher’s exact test explored differences between groups. The Wilcoxon rank sum paired comparison compared QoL scores between parents and patients. The relationship between continuous variables was explored through bivariate correlations and linear regression. A *P* value < 0.05 was considered statistically significant. Statistical analysis and computation were conducted using R (17), SPSS Version 25 and GraphPad Prism 7. SDS for weight, height and BMI were calculated using the Growth Analyser RCT Software based on the WHO data for The United Kingdom of Great Britain and Northern Ireland.

## Results

### Study participants

Of 125 patients with 21OHD aged 8–18 years initially recruited and consented to enter the study, 107 underwent the study visit; 18 patients withdrew or failed to attend the arranged visits. Six patients diagnosed as non-classic 21OHD were excluded from the analysis. Patients were 12.4 (10.3–15.1) years old (median with interquartile range (IQR)) and 53.5% were females. The 83 controls had similar age and sex distribution but differed by ethnicity due to fewer Asian Pakistani controls (*P* < 0.001) (Table 1).

**Table 1:** Demographic characteristics of participants. Data are presented as *n* (%) or as median (range).

	Patients	Controls	*P*-value
Participants, *n*	101	83	
Age (years)	12.4 (10.3–15.1)	13.3 (10.3–15.3)	0.583
<12 years vs 12–18 years			0.574
<12 years	48 (47.5%)	36 (43.4%)	
12–18 years	53 (52.5%)	47 (56.6%)	
Sex			0.336
Girls	54 (53.5%)	45 (54.2%)	
Boys	47 (46.5%)	38 (45.8%)	
Ethnicity			<0.001
White	75 (74.3%)	72 (85.7%)	
Asian Pakistani	20 (19.8%)	0	
Asian other	0	6 (7.2%)	
Black	1 (1.0%)	2 (2.4%)	
Mixed	5 (5.0%)	2 (2.4%)	
Other	0	1 (1.2%)	

### Presentation on diagnosis and patient history

Most patients (68.3%) were diagnosed in the first year of life (Supplementary Fig. 1), presenting with atypical genitalia (34.7%) or salt-losing crisis (25.7%) (Supplementary Table 3). Most patients were born at term (mean: 38.7 (±2.3) weeks gestation). For three patients, the sex assigned at birth was different from the final sex and gender identity (three females that were initially assigned male sex and currently live as females). A family history of CAH was reported in 26.7% of cases. Adrenal crisis requiring admission was documented in 39.6% of patients, of which 12.8% had 3 or more episodes (Supplementary Fig. 2), amounting to 0.2 episodes per patient-year, similar to international data (18). Of the female patients, 61.1% had undergone urogenital examination under anaesthesia and 40.7% had genital surgery (clitoroplasty 33.3%, vaginoplasty 25.9%). Other medical conditions were reported in 26.7% of patients (Supplementary Table 4).

### Glucocorticoid replacement therapy

**Figure 1:**
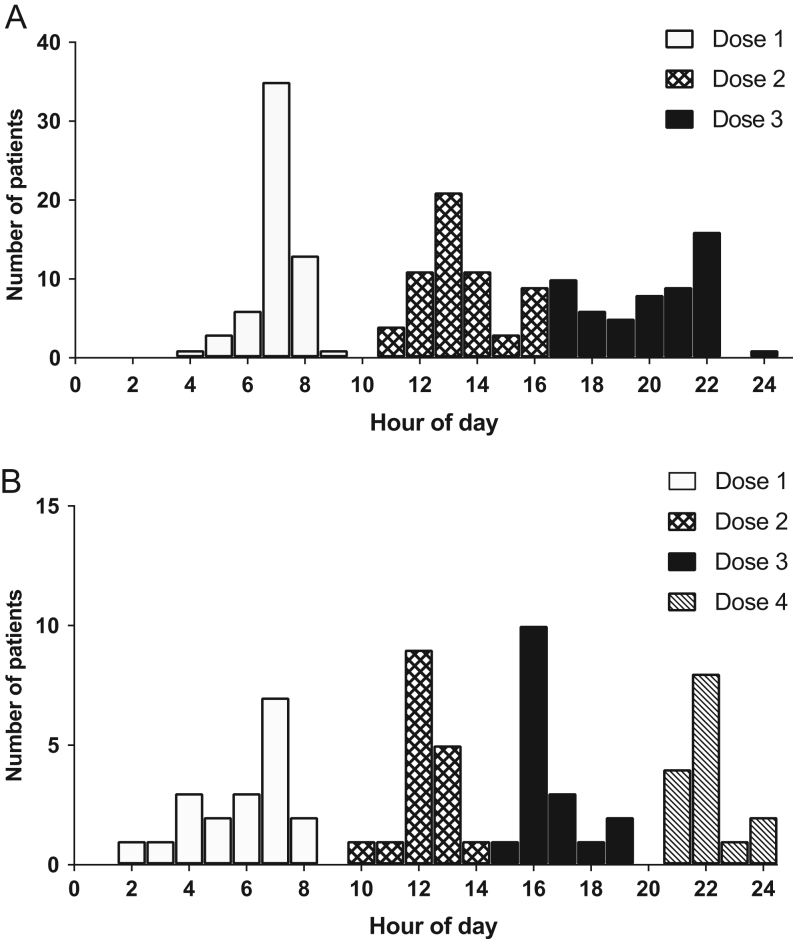
Times of administration of hydrocortisone doses for children on three daily doses (A, *n*  = 61) and children on four daily doses regimes (B, *n*  = 28). Each bar represents the number of patient visits recording a dose given at that time; the different patterns correspond to the order of the doses throughout the day.

The majority of patients (97.0%) were treated with hydrocortisone, six received prednisolone and one with hydrocortisone and prednisolone. Most patients received three (61.7%) or four daily doses of hydrocortisone (28.7%); a small number of patients received two (five patients), five (two patients) and six doses (1 patient). The morning hydrocortisone dose was the highest dose of the day in 53.1% of patients, while a reverse circadian regime was used by 10.6%, administration times varying among patients (Fig. 1). Most patients treated with prednisolone received two daily doses (07:00–08:00 h and 21:00–22:00 h), and one patient received one dose.

**Figure 2:**
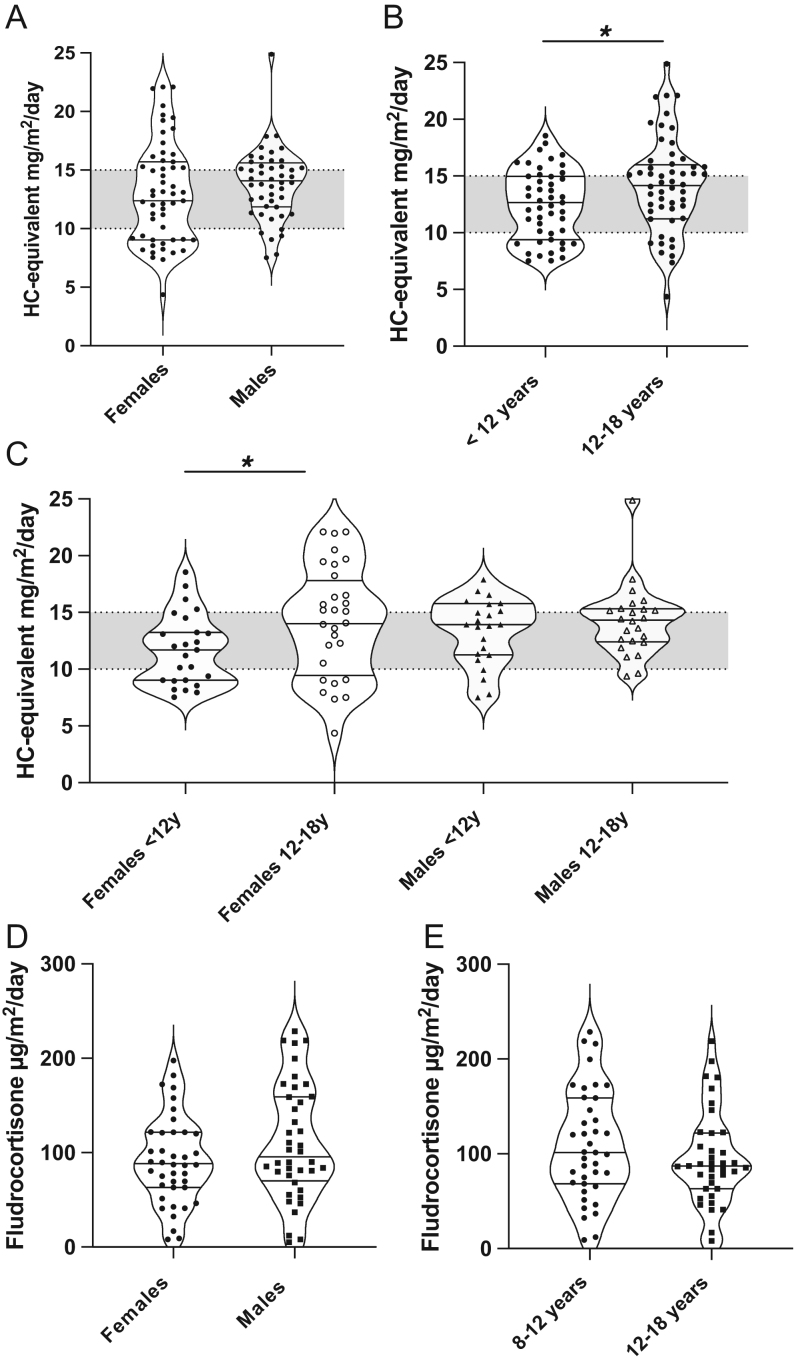
Daily glucocorticoid doses for sex (A) and age (B) groups, as well as subgroups of age and sex (C). The shaded area corresponds to the recommended glucocorticoid dose range of 10–15 mg/m^2^/day hydrocortisone equivalent (2). Daily mineralocorticoid doses for sex (D) and age (E) groups, expressed as fludrocortisone µg/m^2^/day. The horizontal lines correspond to the median, 25th and 75th quartiles (*indicates statistical significance).

The mean relative daily dose was 13.6 (±3.6) mg/m^2^/day hydrocortisone – equivalent (dose range: 7.3–24.8 mg/m^2^/day). Subgroup analysis showed higher relative doses in 12- to 18-year-old females (*P* = 0.008), who had a wider dose range compared to the other subgroups (Fig. 2A, B, C and Table 2). Overall, 47.9% of patients received a daily dose of hydrocortisone within the recommended range of 10–15 mg/m^2^/day (4), while 22.3% of daily doses were below and 29.8% above this range. Doses below 10 mg/m^2^/day were more frequently used in females compared to males (*P* **= 0.023); these cases were evenly distributed between centres. We found no relationship between the timing of doses and the adherence to the recommended dose range.

**Table 2:** Daily glucocorticoid and mineralocorticoid doses for age and sex groups and subgroups. Glucocorticoid doses are expressed as hydrocortisone-equivalent mg/m^2^/day and mineralocorticoid doses as fludrocortisone µg/m^2^/day and µg/day. The subgroup comparison was conducted for the relative doses for both GC and fludrocortisone. Daily doses are expressed as mean ± s.d. (*n*  = number of participants included in the analysis).

Group	*n*	Daily dose	Subgroup comparison			
			Males vs females	8–12 vs12–18 years	8–12 vs12–18 years	
					Males	Females
HC-equivalent (mg/m^2^/day)			0.544	0.007	0.373	0.008
All	101	13.6 ± 3.6				
Males	47	13.8 ± 2.9				
Females	54	13.3 ± 4.2				
Age, years						
8–12 years	48	12.5 ± 3.0				
12–18 years	53	14.5 ±3.9				
Fludrocortisone			0.231	0.188	0.063	0.808
All	82					
µg/m^2^/day		105.6 ± 49.3				
µg/day		154.3 ± 70.1				
Males	40					
µg/m^2^/day		115.1 ± 54.8				
µg/day		169.8 ± 70.7				
Females	42					
µg/m^2^/day		96.5 ± 42.1				
µg/day		139.6 ± 67.0				
Age, years						
8–12 years	39					
µg/m^2^/day		114.0 ± 52.9				
µg/day		145.9 ± 64.1				
12–18 years	43					
µg/m^2^/day		98.3 ± 45.3				
µg/day		161.6 ± 74.8				

### Mineralocorticoid replacement therapy

Mineralocorticoid replacement was reported in 82 patients (81.8%). Fludrocortisone was administered once daily in 85.4% of cases, most frequently at 08:00 h. Total fludrocortisone daily doses ranged between 25 and 350 µg/day, while relative doses ranged between 16.8 and 228.6 µg/m^2^/day with a mean of 105.6 (±49.3) µg/m^2^/day. There were no significant differences in relative doses between age and sex subgroups (Fig. 2D, E, Table 2 and Supplementary Fig. 3).

### Biochemical markers of control

**Figure 3:**
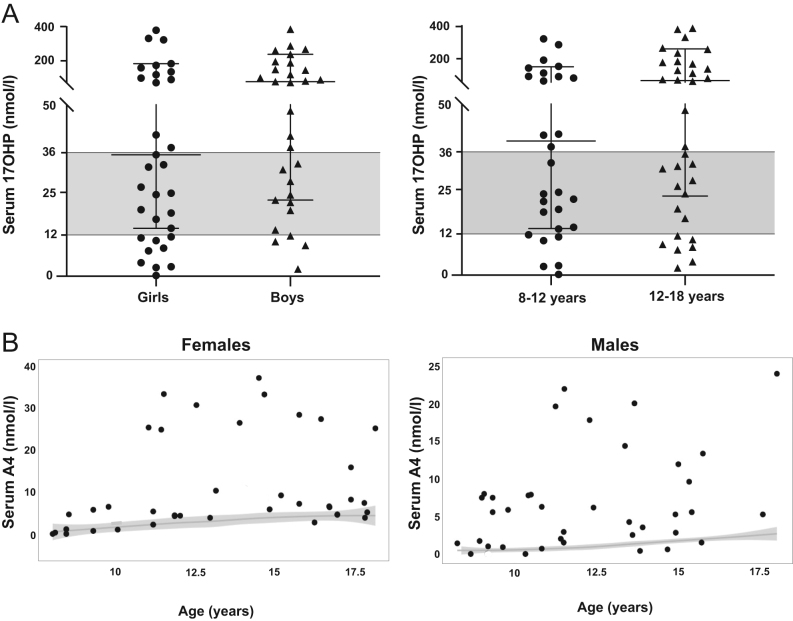
(A) Concentration of serum 17-hydroxyprogesterone in CAH patients for sex and age groups. The shaded areas correspond to the recommended range for hormone concentrations in CAH (12). (B) Concentration of serum androstenedione (A4) in gender groups; the grey ribbons represent the 95% CI of the linear regression of the normal serum A4 ranges with age (13).

Morning androstenedione concentrations were elevated in 68.6% and low in 3.5% of patients. Plasma 17-hydroxyprogesterone was below the recommended range (12–36 nmol/L (13)) suggesting suppression in 13.5% of patients (Fig. 3). Concentrations were similar between sex and age groups, except for androstenedione (Chi-square *P* **= 0.038), males having more values above normal range than females (81% vs 55%). Relative GC doses did not correlate with plasma androstenedione or 17-hydroxyprogesterone, with no significant difference in these biomarkers based on GC dose.

### Clinical and anthropometric characteristics

The comparison of pubertal development between patients and controls, assessed by Tanner staging, showed advanced stages of pubarche in females (*P *= 0.010) and genital development in males (*P *= 0.017) with CAH (Supplementary Fig. 4).

**Figure 4:**
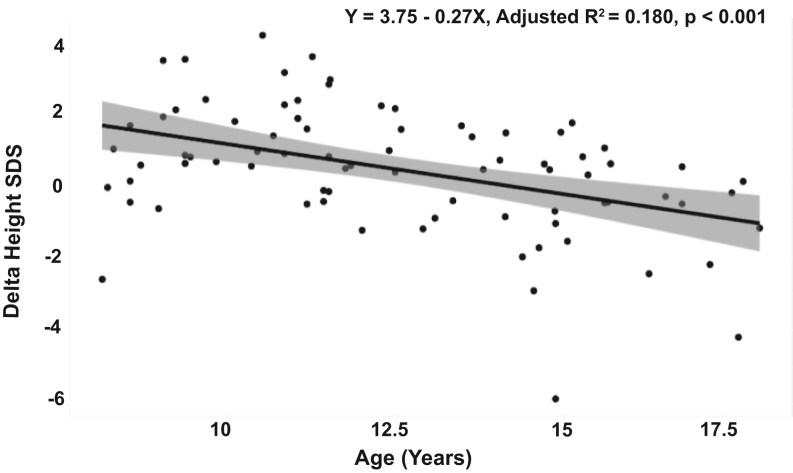
Anthropometric characteristics in patients with CAH. Linear regression showing the relationship between delta height SDS (difference between height SDS and mid-parental height SDS in patients with CAH).

Regression analysis showed delta height SDS decreasing with age, indicating that the discrepancy between achieved height and genetic potential height increases as patients reach adulthood (Fig. 4). Compared to controls, 8- to 12-year-old patients were taller (*P *= 0.015) and patients aged 12–18 years were shorter (*P *= 0.035), regardless of the age on diagnosis and duration of GC treatment (Table 3). CAH patients had higher weight SDS (*P *= 0.022), BMI SDS (*P *= 0.002), waist SDS (*P *= 0.002) and hip circumference SDS (*P *= 0.009) than controls (Table 3). Subgroup analysis demonstrated a significantly increased BMI only in female patients (*P *= 0.007) and patients aged 12–18 years (*P *= 0.011). Waist circumference SDS were above controls in female patients (*P *= 0.009) and patients over 12 years (*P *= 0.010), while hip circumference was higher in patients only for males aged 8–12 years (*P *= 0.024). Based on BMI SDS, we found that 27% of CAH patients were overweight and 22% obese, compared to 10.8% and 10.8%, respectively, in controls (*P *< 0.001), and the statistical difference was maintained for age and sex subgroups. Patients receiving relative hydrocortisone doses below 10 mg/m^2^/day had higher weight SDS (*P *= 0.008) and BMI SDS (*P *= 0.007) compared to the rest of the cohort. Regression analysis showed no relationship between relative hydrocortisone dose and anthropometric measurements. There was no significant difference in relative GC doses between obese/overweight and normal-weight patients. (Supplementary Fig. 5). There was no correlation between BMI and plasma androstenedione and 17-hydroxyprogesterone.

**Table 3:** Anthropometric data comparison patients vs controls. The variables are expressed as median with interquartile ranges of SDS scores.

Variable	Patients		Controls		*P*-values	Subgroup comparison			
	*n*	Values	*n*	Values	Patients vs controls	Males		Females	
						8–12 years	12–18 years	8–12 years	12–18 years
Height						0.030	0.188	0.348	0.125
All	101	0.3 (−0.7 to 1.2)	83	0.17 (−0.4 to 0.8)	0.742				
Males	47	0.4 (−0.8 to 2.2)	38	0.06 (−0.6 to 1.5)	0.328				
Females	54	0.2 (−0.7 to 0.8)	45	0.2 (−0.3 to 0.7)	0.768				
Age									
8–12 years	48	0.8 (0.1–2.3)	36	0.1 (−0.3 to 1.2)	0.015				
12–18 years	53	−0.3 (−1.1 to 0.5)	47	0.1 (−0.5 to 0.6)	0.035				
Weight						0.114	0.783	0.123	0.200
All	101	0.9 (0.0–1.8)	83	0.2 (−0.4 to 1.0)	0.022				
Males	48	1.2 (0.1–2.0)	38	0.3 (−0.2 to 1.2)	0.092				
Females	59	0.5 (0.1–1.7)	45	0.1 (−0.5 to 0.7)	0.049				
Age									
8–12 years	48	1.3 (0.3–2.2)	36	0.5 (−0.4 to 1.4)	0.024				
12–18 years	53	0.6 (−0.5 to 1.6)	47	0.1 (−0.3 to 0.6)	0.343				
BMI						0.483	0.149	0.152	0.013
All	101	1.0 (−0.1 to 1.9)	83	0.2 (−0.3 to 0.8)	0.002				
Males	48	1.1 (−0.2 to 2.0)	38	0.3 (−0.1 to 1.0)	0.088				
Females	59	0.7 (−0.0 to 1.9)	45	0.0 (−0.4 to 0.6)	0.007				
Age									
8–12 years	48	1.0 (−0.2 to 2.2)	36	0.4 (−0.3 to 1.0)	0.110				
12–18 years	53	1.0 (−0.1 to 1.8)	47	0.1 (−0.3 to 0.8)	0.011				
Waist circumference						0.336	0.216	0.791	0.031
All	91	1.2 (0.4–2.3)	78	0.7 (−0.1 to 1.4)	0.002				
Males	45	1.1 (0.4–2.2)	34	0.7 (−0.1 to 1.4)	0.098				
Females	46	1.2 (0.4–2.3)	44	0.6 (−0.1 to 1.2)	0.009				
Age									
8–12 years	44	1.1 (0.4–2.3)	33	0.9 (−0.2 to 1.4)	0.078				
12–18 years	47	1.3 (0.4–2.0)	45	0.6 (−0.1 to 1.1)	0.010				
Hip circumference						0.024	0.128	0.779	0.222
All	93	0.9 (−0.6 to 1.7)	78	0.0 (−1.0 to 0.8)	0.009				
Males	46	1.3 (−0.3 to 2.4)	34	0.1 (−0.6 to 1.2)	0.015				
Females	47	0.5 (−0.8 to 1.3)	44	0.0 (−1.0 to 0.8)	0.243				
Age									
8–12 years	44	1.0 (−0.4 to 1.7)	33	0.0 (−0.6 to 1.1)	0.044				
12–18 years	49	0.7 (−0.7 to 1.7)	47	0.0 (−1.0 to 0.7)	0.085				

Raised blood pressure was found in five patients (three females, two males) and one control (female). Of these, four received fludrocortisone in doses between 60 and 200 µg/m^2^/day. Their GC doses ranged between 9 and 15 mg/m^2^/day hydrocortisone. One of these patients was overweight and one had cushingoid features.

### Bone age

Bone age results were available for 86 CAH patients. The difference between bone age and chronological age was 1.8 (±2.3) years. Bone age was advanced by over 1.5 years in 40.5% of patients and delayed by over 1.5 years in 4 patients. There was no significant difference between the sexes. We found no correlation between bone age and relative GC doses and no significant distinction between different dose groups.

### Biochemical markers of metabolic risk

For most patients, the results of non-steroid serum biochemistry were within normal limits (Supplementary Table 5). A third of the patients had raised plasma renin or renin activity; however, their gluco- and mineralocorticoid doses were similar to those of patients with normal values. A small proportion of patients had altered lipid profiles with raised total cholesterol (3.0%), low HDL (3.0%), raised LDL (7.0%) and triglycerides (5.0%). Insulin resistance defined by HOMA-IR was found in 53.2% of CAH patients, similar to controls (57.7%) across age, sex and dose subgroups. There was no difference in GC doses between patients with high and normal HOMA-IR.

### Quality-of-life assessment

Questionnaires were completed by 101 patients and 98 parents. In the PedsQL questionnaires, ‘school functioning’ scored lowest, 70 (55–80) median (IQR), followed by ‘emotional functioning’ 75 (65–85), ‘physical functioning’ 87 (75–93) and ‘social functioning’ 95 (81–100). PedsQL scores correlated well between patients and parents (*r _s_=* 0.5, *P *< 0.001). Wilcoxon rank-sum comparison showed that parents scored their offspring lower than patients scored themselves; however, there was no statistically significant difference (Supplementary Fig. 6). SDQ results also correlated highly between patients’ and parents’ scores across all domains (Pearson’s rank correlation *P *< 0.05). Total SDQ scores placed 16.3% of patients within the ‘high’ and ‘very high’ categories of concern (warranting mental health assessment), the worst scores were noted in the ‘hyperactivity’ domain where 14.1% raised ‘very high’ concerns (Supplementary Fig. 7). In females, the QoL scores were not significantly different between patients who had undergone genital surgery and those who had not.

## Discussion

In this study, we provide the first large national overview of the health status of children and young persons with CAH, derived from a standardised assessment of clinical and biochemical parameters, as well as based on QoL questionnaires. While in adults with CAH the raised prevalence of comorbidities including metabolic and cardiovascular disease is well established (1), we found that in children growth and weight gain problems are the most common health-related issues. However, some of the findings would suggest that the onset of metabolic comorbidities may take place during childhood and adolescence.

Growth patterns in children with CAH differed from healthy controls, being accelerated before puberty and indicating reduced post-pubertal height. The age-dependent fluctuation of patient height in relation to the mid-parental height showed reduced final stature in relation to the genetic potential. Patients aged 8–12 years were taller than controls with advanced bone age and higher Tanner scores, consistent with the higher levels of adrenal androgens responsible for the early growth spurt in CAH. Previous evidence from the UK showed reduced final height in adults with CAH (19), and our results indicate that children are still at risk of impaired height outcomes. A third of the patients exceeded the recommended dose range of 10–15 mg/m^2^/day HC equivalent (4) and the reduced height in older patients may also relate either to the growth-suppressive effect of GCs. While the relative GC doses did not correlate with height SDS in our cohort, a previous longitudinal study found a significant correlation between doses and final height, reporting an increased prevalence of short stature for hydrocortisone doses over 17 mg/m^2^/day (20).

The proportion of overweight or obese patients was higher compared to controls. Overweight and obesity were less prevalent in our control group compared to national data reporting variations between 11% and 29% in year-6 children, depending on the socio-economic status (21); thus, our results may to some extent overestimate the impact of CAH on weight gain. Nevertheless, our findings are consistent with the UK data from adults with CAH (19) and with studies conducted in other countries on children with CAH (22, 23). Only one patient in our cohort was reported to have cushingoid features, compared to 63% of females and 38% of males reported in adults (19). This might relate to increased awareness of GC toxicity or to a shorter duration of exposure than adults. The regression analysis found no significant relationship between weight/BMI and GC doses; however, in the subgroup of overweight patients, the doses were lower compared to those with normal BMI. This could be explained by variations in the 11beta-hydroxysteroid dehydrogenase activity in adipose tissue, but also raises the question of whether GC dose should be prescribed for the lean body mass

Identifying raised blood pressure was based on single time-point assessments, insufficient to diagnose hypertension (16), and only found in five patients which prevented us from exploring predisposing factors. The impact of CAH on blood pressure remains unclear, especially in children. While there are studies showing raised blood pressure in children with CAH (23, 24), the overall evidence is controversial (25), with one study even reporting hypotension in normal-weight patients (26).

Altered lipid profiles were found in a modest number of patients in comparison to adult data (19); however, the significant prevalence of overweight and obesity among our cohort indicates an increased risk of developing metabolic syndrome. This is supported by evidence showing the raised prevalence of the metabolic syndrome before puberty (27). We identified insulin resistance in 53.2% of patients, much higher than the 28–36% prevalence reported in adults with CAH (19). This may relate to using lower HOMA-IR thresholds in children (14). There are discrepancies in the published evidence related to the prevalence of IR in children with CAH compared to controls (23, 28, 29). Our study could not confirm the development of IR during childhood due to CAH, the raised HOMA-IR in all participants being likely linked to the rising prevalence of metabolic syndrome in the general paediatric population (30).

GC replacement mainly consisting of hydrocortisone administered in three or four daily doses is in keeping with the international recommendations based on the fast clearance of hydrocortisone, aiming to mimic physiological cortisol profiles (4). Evidence comparing the benefits of circadian and reverse circadian administration regimes has so far been inconclusive in CAH (31); however, in adrenal insufficiency, evening elevation of plasma cortisol was associated with an increased risk of metabolic complications (32). The hydrocortisone dose was within the recommended range (10-15 mg/m^2^/day) in less than half of the patients, with lower doses used in 22.3% and higher doses in 29.8% of patients. This corresponded to a higher frequency of doses below 10 mg/m^2^/day compared to global data (5), and we observed that female patients aged 12–18 years had a wider range of GC replacement compared to the other subgroups. This finding could not be linked to other hormonal treatments or a particular centre and has not been previously reported. Arguably, it could relate to differences in compliance, especially since androstenedione was more frequently elevated in male patients compared to females. Patients aged 12–18 years had significantly higher doses, despite similar concentrations of androstenedione and 17-hydroxyprogesterone. This might suggest either decreased compliance or increased GC requirement, due to changes in hydrocortisone pharmacokinetics as children get older, attributed to variations in the 11β-hydroxysteroid dehydrogenase activity (33). Hormonal measurements were conducted between 08:00 and 10:00 h after patients had taken the initial GC dose at variable hours, which may have affected their value in monitoring treatment. Moreover, the limitations of current biochemical markers of control are well known (34), and superior monitoring tools are still to be developed. Thus, our study highlights the need to individualise the interpretation of hormonal biomarkers in CAH (4).

Mineralocorticoid therapy involved a wide range of fludrocortisone doses in age and sex subgroups, most likely related to variations in clinical practice. A third of the patients had raised plasma renin/ renin activity, suggesting insufficient mineralocorticoid replacement. Consideration must be given to the regional variation in the type of assay used. However, previous evidence showed no correlation between plasma renin and either blood pressure or mineralocorticoid doses in adults and children (35). This suggests that renin may not be a sensitive marker for detecting under- and over-treatment, indicating that better monitoring tools are needed to guide fludrocortisone treatment.

Our work also highlights significant problems related to the QoL and the psychological health of children with CAH. QoL was significantly impaired in a large proportion of patients; however, there was variability across included subjects. Based on the SDQ scores, a psychiatric assessment would be indicated in 16.3% of the cohort. PedsQL scores were lower for parents’ assessments compared to their children’s, implying a different perception of the impact of their condition; this indicates a need to actively engage patients and parents in the dialogue with multidisciplinary team professionals. School functioning, the lowest scoring category in the PedsQL, may be explained by reduced cognitive function (36, 37) or mental health issues (4). An early diagnosis associates with better cognitive outcomes (38), a potential explanation being a reduced risk of hypoglycaemia and adrenal crisis. Changes in the function and structure of the brain in CAH have been evidenced through MRI (39), although their direct mechanism remains unclear. Overall, the data derived from QoL questionnaires suggest the need for psychology support for children with CAH as part of the multidisciplinary team, as is currently the practice for type 1 diabetes and other chronic conditions.

One limitation of our study consisted of the number of participants, which was smaller than we had expected based on the UK prevalence of 21OHD and previous participation in research from CAH patients and families. Despite that, we consider that our prospective cohort of 101 CAH patients provides a comprehensive overview of the health status of children with CAH in the UK. Another potential study weakness related to our control group which was not matched to patients ethnically and socio-economically. The controls’ prevalence of overweight and obesity appeared to be below the national benchmark, possibly due to reduced participation from the more deprived population groups. However, as discussed, our findings related to abnormal growth patterns and increased weight in CAH are consistent with previous evidence, including data from adult patients. A potential study limitation is the absence of matched control data for the QoL analysis, where we used national normative data for SDQ, while for the PedsQL scores we undertook subgroup analysis.

Overall, our work emphasises the need for an expert multidisciplinary approach when managing children with CAH, including endocrine specialist follow-up and psychologist input, to help limit the development of comorbidities and improve QoL. Moreover, standardised data collection for auditing health care provision and health status should be implemented to further explore and understand the health problems highlighted by this study.

## Supplementary Material

Supplementary Material

Supplementary Table 1. List of collaborating centers

Supplementary Table 2. Inclusion and exclusion criteria for patients and controls

Supplementary Table 3. Reason for initial presentation

Supplementary Table 4. Medical background

Supplementary Table 5. The results of biochemical investigations in patients with CAH in relation to the normal ranges of the respective local laboratories.

Supplementary Figure 1. Age on diagnosis

Supplementary Figure 2. Frequency of episodes of adrenal crisis requiring admission (n=35)

Supplementary Figure 3 Daily absolute mineralocorticoid doses for sex (A) and age (B) groups, expressed as fludrocortisone µg/day. The horizontal lines correspond to the median, 25th and 75th quartiles. (*indicates statistical significance)

Supplementary Figure 4. Pubertal development assessed by Tanner Stages in girls and boys, comparison between patients (black) and controls (white).

Supplementary Figure 5. Comparison of weight (A), height (B) and BMI (C) standard deviation scores (SDS) between patient subgroups of relative GC daily dose. Patients were divided between those receiving < 10, 10-15 and > 15 mg HC-equivalent/m2/day. (D) Comparison of relative daily GC doses between patients with normal BMI and patients with BMI > 2SD, classified as overweight or obese. (*indicates statistical significance)

Supplementary Figure 6. Paediatric Quality of life total scores in patients and parents (violin plots) compared to normative data, courtesy of Varni, et al. 2007. (boxes with error bars indicate mean with standard deviation)

Supplementary Figure 7. SDQ scores in patients and parents, classified in categories of concern, compared to normative population data.

## Declaration of interest

This study has been financially supported by the National Institute of Health Research rare disease translational research collaboration (NIHR RD TRC), the Chief Scientist Office of Scotland and Diurnal Ltd.

## Funding

This study was financially supported by the National Institute of Health Research rare disease translational research collaboration (NIHR RD TRC), Nils P Krone.

## Author contribution statement

N K and S F A were responsible for the study conceptualisation and design. N K wrote all the protocol versions, obtained ethical approval and funding. S M was the project administrator. S A, T C, E C, U D, M D, J H D, E G, R K, L P, T R, F R, S F A and N K coordinated the study sites, including participant recruitment. I B, S M, S A, T C, E C, U D, M D, J H D, E G, R K, A K, L P, T R, F R, S F A and N K were responsible for data collection, including clinical data and biological samples. B K conducted the centralised measurement of blood steroid hormones by liquid chromatography tandem mass spectrometry. I B and N L conducted the data analysis. I B, N L and N K wrote the first draft of the manuscript and all authors contributed to data interpretation, manuscript revision and editing.
