# Supplementary material for: Health status of children and young persons with congenital adrenal hyperplasia in the UK (CAH-UK): a cross-sectional multi-centre study
Source: Eur J Endocrinol. 2022 Aug 24;187(4):543–53. doi: 10.1530/EJE-21-1109 (PMC9513639; doi:10.1530/EJE-21-1109)
Supplement: Supplementary Material [file supplementary_material.pdf]

Health Status of Children and Young Persons with Congenital Adrenal Hyperplasia in the UK (CAH-UK)

Supplementary file 1

Case Record Form – CAH Patients

CAH – UK

Health status in CYPs with CAH

Case Record Form

Cross-sectional study of children and young person with CAH in the UK

Patient Name ______________________________

Patient NHS Number ______________________________

Patient Hospital Number ______________________________

Patients Study Number ______________________________

Has the patient signed the consent form and been given a copy of the patient information sheet?

☐ YES ☐ NO

Has the patient been registered in the I-CAH?

☐ YES ☐ NO

Please tick only one box per question unless otherwise instructed.

This page should be detached and kept with a copy of the consent form at the local research centre

PATIENT'S DETAILS

1. Patient's Study Number ______________________________

2. Date seen in clinic ______________________________

3. Age seen in clinic decimal ____________ yr/ months ____________

4. Sex ☐ Male ☐ Female

5. Ethic origin

☐ White

☐ Mixed
- ☐ White and Black Caribbean
- ☐ White and Black African
- ☐ White and Asian
- ☐ Other, details………………………………

☐ Asian
- ☐ Indian
- ☐ Pakistani
- ☐ Bangladeshi
- ☐ Other, details………………………………

☐ Black
- ☐ Caribbean
- ☐ African
- ☐ Other, details………………………………

☐ Chinese

☐ Any other ethic group, details…………………………………

MEDICAL HISTORY (1)

6. Reason for initial presentation (please tick all that apply)

☐ Prenatal diagnosis ☐ Atypical genitalia ☐ Tall stature

☐ Salt losing crisis ☐ Early puberty ☐ Family screening

☐ Other, please specify……………………………………………………………………………………

7. Age at presentation ________ years ________ months ________ days

XX. Birth weight ________ grams ________ lb ________ oz

XX. Birth length ________ cm

8. Gender assignment at birth

☐ male ☐ female

☐ Not assigned ☐ unknown

9. Family History of CAH

☐ YES ☐ NO

If YES, please provide details of type and family connection

10. Other significant medical history

11. Adrenal crisis after first diagnosis

☐ YES ☐ NO

If yes, how often, specify hospital admissions

MEDICAL HISTORY (2)

Parental height (use either units – cm if measured)

XX. Fathers's height ________ cm ________ ft ________ inches

XX. Mother's height ________ cm ________ ft ________ inches

If female

Prader Genital stage ________

Examination under anaesthesia (EUA) ☐ YES ☐ NO ☐ Not known

Genital surgery ☐ YES ☐ NO ☐ Not known

If YES, please provide details of type and date of surgery

Clitoral ☐ YES ☐ NO Date(s) ________________

____

____

Vaginal ☐ YES ☐ NO Date(s) ________________

____

____

Age at menarche (years) ________

Menarche ☐ Spontaneous ☐ Induced

Regular menstrual cycle ☐ YES ☐ NO

MEDICATION

Glucocorticoid type (please tick all that apply)

☐ Hydrocortisone ☐ Prednisolone ☐ Dexathmethasone

☐ Other or combinations, please specify……………………………………………………………

Glucocorticoid preparation (please tick all that apply)

☐ Tables ☐ Corlan pellets ☐ Dissolved tablets

☐ Premade Solution ☐ Other, please specify……………………………………

Glucocorticoid dose

Dose (in mg) ________ - ________ - ________ - ________

time ________ ________ ________ ________

Fludrocortisone replacement

Dose (in µg) ________ - ________ - ________ - ________

time ________ ________ ________ ________

Salt replacement

Dose (in mmol per dose) ________ Times daily ________

**Glucocorticoid doses based on BSA?**	☐ YES	☐ NO	☐ Not known
**Does patient own a steroid card?**	☐ YES	☐ NO	☐ Not known
**Does patient own medic alert ID?**	☐ YES	☐ NO	☐ Not known
**Steroid card with her/ him today?**	☐ YES	☐ NO	☐ Not known

Other Medication

**Oestrogen**	☐ YES	☐ NO	☐ Not known
**Testosterone**	☐ YES	☐ NO	☐ Not known
**GnRH agonist**	☐ YES	☐ NO	☐ Not known
**Antihypertensive**	☐ YES	☐ NO	☐ Not known
**Antidiabetic**	☐ YES	☐ NO	☐ Not known
**Antidepressant**	☐ YES	☐ NO	☐ Not known
**Other**	☐ YES	☐ NO	☐ Not known

PHYSICAL EXAMINATION

Auxology

Height (in cm) ____________ **Waist circumference (in cm)** ____________

Weight (in kg) ____________ **Hip circumference (in cm)** ____________

Blood pressure

	Systolic		Diastolic	
**Sitting 1**	____________	/	____________	mmHg
**Sitting 2**	____________	/	____________	mmHg
**Sitting 3**	____________	/	____________	mmHg
**Standing**	____________	/	____________	mmHg

Pubertal status

Pubic hair stage ____________ Breast stage (females) ____________

Axillary hair stage ____________ Genital stage (males) ____________

If male

Testicular volume Left ________ (ml) Right ________ (ml)

Testicular masses on palpation ☐ YES ☐ NO ☐ Not known

Cushingoid ☐ YES ☐ NO ☐ Not known

Virilisation ☐ YES ☐ NO ☐ Not known

If YES, please provide details of cushingoid features or signs of virilisation

BIOCHEMICAL ANALYSIS

To be performed after overnight fast. Ice must be available for ACTH samples, adipokine samples and samples for metabolomics analysis.

Date of sample ____________ **Time of sample** ____________

Time of last glucocorticoid dose ____________

17OHP profile (most recent/ latest to visit) please cross out if not done

**Time**				
**Blood spot**				
**Saliva**				

Routine blood analysed in local laboratory (annual review)

	Test	Result
Urea & electrolytes	Sodium	mmol/L
	Potassium	mmol/L
	Urea	mmol/L
	Creatinine	mmol/L
	Plasma renin/ Renin activity	U/L or pg/ml/h
Hormones	Testosterone	nmol/l
	Androstenedione	nmol/l
	17-OH Progesterone	nmol/l
Metabolic	Fasting HDL	mmol/l
	Fasting LDL	mmol/l
	Fasting Triglycerides	mmol/l
	Fasting Cholesterol	mmol/l
	Fasting glucose	mmol/l

**Blood taken for Steroid Analysis (LC-MS/MS)**	☐ YES	☐ NO
**Blood taken for Alternative Steroid (LC-MS/MS)**	☐ YES	☐ NO
**Blood taken for ACTH**	☐ YES	☐ NO
**Blood taken for Adipokine analysis**	☐ YES	☐ NO
**Blood taken for Insulin and C-peptide**	☐ YES	☐ NO
**Blood taken for Metabolomic analysis**	☐ YES	☐ NO
**Saliva taken for steroid analysis (LC-MS/MS)**	☐ YES	☐ NO
**24-hour urine for GC/MS**	☐ YES	☐ NO

ADDITIONAL INFORMATION

Bone age (please tick all that apply)

TW20 ☐ years ______

TW3 ☐ years ______

RUS ☐ years ______

G&P ☐ years ______

Date of bone age ______

Results of 21-hydroxylase (*CYP21A2*) gene analysis

	Mutation1	Mutation2
**Protein level***		
**DNA level***		

*Mutation on either protein or DNA level will be sufficient

Parents tested? ☐ YES ☐ NO ☐ Not known

Additional details

Please give any additional details here that you feel are of note to the study

CAH – UK

Health status in CYPs with CAH

Case Record Form -CONTROLS

Cross-sectional study of children and young person with CAH in the UK

Control Name ______________________

Control NHS Number ______________________

Control Hospital Number ______________________

Control Study Number ______________________

Has the patient signed the consent form and been given a copy of the patient information sheet?

☐ YES ☐ NO

Please tick only one box per question unless otherwise instructed.

This page should be detached and kept with a copy of the consent form at the local research centre

CONTROLS'S DETAILS

1. Control Study Number ______________________

2. Date seen in clinic ______________________

3. Age seen in clinic decimal ________ yr/ months ________

4. Sex ☐ Male ☐ Female

5. Ethic origin

☐ White

☐ Mixed
- ☐ White and Black Caribbean
- ☐ White and Black African
- ☐ White and Asian
- ☐ Other, details……………………………

☐ Asian
- ☐ Indian
- ☐ Pakistani
- ☐ Bangladeshi
- ☐ Other, details……………………………

☐ Black
- ☐ Caribbean
- ☐ African
- ☐ Other, details……………………………

☐ Chinese

☐ Any other ethic group, details………………………………

Control study number

MEDICAL HISTORY

Exclusion criteria checked?

**Past or present history of an endocrinopathy (all stages)?**	☐ YES	☐ NO
**Type 1, diabetes, Type 2 diabetes, Insulin resistance?**	☐ **YES**	☐ **NO**
**Known conditions of lipid/ cholesterol metabolism?**	☐ YES	☐ NO
**Pregancy?**	☐ YES	☐ NO
**Presence of any psychiatric disorder?**	☐ YES	☐ NO
**Current or past use of psychiatric medication?**	☐ YES	☐ NO
**Glucocorticoid use within the last 6 months?**	☐ YES	☐ NO
**Diagnosed learning difficulties and/or full-scale IQ <70?**	☐ YES	☐ NO
**Medication known to effect steroid metabolism?**	☐ YES	☐ NO

Please note, if any question answered with YES, individual is not eligiable to be recruited as control.

PHYSICAL EXAMINATION

Auxology

Height (in cm) ______________ **Waist circumference (in cm)** ______________

Weight (in kg) ______________ **Hip circumference (in cm)** ______________

Blood pressure

	**Systolic**		**Diastolic**	
**Sitting 1**	______________	/	______________	mmHg
**Sitting 2**	______________	/	______________	mmHg
**Sitting 3**	______________	/	______________	mmHg
**Standing**	______________	/	______________	mmHg

Pubertal status (self-reported)

Pubic hair stage ______________ Breast stage (females) ______________

Axillary hair stage ______________ Genital stage (males) ______________

BIOCHEMICAL ANALYSIS

To be performed after overnight fast. Ice must be available for samples for adipokine analysis.

Date of sample ____________ **Time of sample** ____________

**Fasting glucose**	mmol/l

**Blood taken for Steroid Analysis (LC-MS/MS)**	☐ YES	☐ NO
**Blood taken for Alternative Steroid (LC-MS/MS)**	☐ YES	☐ NO
**Blood taken for ACTH**	☐ YES	☐ NO
**Blood taken for Adipokine analysis**	☐ YES	☐ NO
**Blood taken for Insulin and C-peptide**	☐ YES	☐ NO
**Saliva taken for steroid analysis (LC-MS/MS)**	☐ YES	☐ NO
