# Supplementary material for: Health status of children and young persons with congenital adrenal hyperplasia in the UK (CAH-UK): a cross-sectional multi-centre study
Source: Eur J Endocrinol. 2022 Aug 24;187(4):543–53. doi: 10.1530/EJE-21-1109 (PMC9513639; doi:10.1530/EJE-21-1109)
Supplement: Supplementary Table 1. List of collaborating centers [file supplementary_table_1.pdf]

Supplementary Table 1. List of collaborating centers

1	Sheffield Children's Hospital and NIHR Clinical Research Facility, Sheffield Teaching Hospital, Sheffield
2	Birmingham Children's Hospital, Birmingham
3	Royal Hospital for Sick Children, Yorkhill, Glasgow
4	Great Ormond Street Hospital, London
5	Addenbrooke's Clinical Research Centre, Cambridge
6	Bristol Royal Hospital for Children, Bristol
7	Royal Manchester Children's Hospital, Manchester
8	Oxford Children's Hospital, Oxford
9	Alder Hey Children's Hospital, Liverpool
10	Great North Children's Hospital, Newcastle
11	Leeds General Infirmary, Leeds
12	University Hospital Southampton, Southampton
13	The Royal London Hospital, London
14	Nottingham University Hospitals NHS Trust, Nottingham
