# Supplementary material for: Health status of children and young persons with congenital adrenal hyperplasia in the UK (CAH-UK): a cross-sectional multi-centre study
Source: Eur J Endocrinol. 2022 Aug 24;187(4):543–53. doi: 10.1530/EJE-21-1109 (PMC9513639; doi:10.1530/EJE-21-1109)
Supplement: Supplementary Table 2. Inclusion and exclusion criteria for patients and controls [file supplementary_table_2.pdf]

Supplementary Table 2. Inclusion and exclusion criteria for patients and controls

**Patients**	
**Inclusion criteria**	**Exclusion criteria**
• Known diagnosis of 21OHD confirmed by hormonal or genetic testing<br>• Age between eight and 18 years<br>• Capacity to consent/assent and provide a signed and dated informed consent	• Pregnancy
**Controls**	
**Inclusion criteria**	**Exclusion criteria**
• Age between eight and 18 years<br>• Capacity to consent/assent and provide a signed and dated informed consent	• Past or present history of an endocrinopathy (all stages)<br>• Type 1, diabetes, type 2 diabetes, insulin resistance<br>• Known conditions of lipid/ cholesterol metabolism<br>• Presence of any psychiatric disorder, current or past use of psychiatric medication<br>• Glucocorticoid use within the last 6 months<br>• Diagnosed learning difficulties and/or full-scale IQ <70<br>• Medication known to effect steroid metabolism<br>• Pregnancy
