# Supplementary material for: Health status of children and young persons with congenital adrenal hyperplasia in the UK (CAH-UK): a cross-sectional multi-centre study
Source: Eur J Endocrinol. 2022 Aug 24;187(4):543–53. doi: 10.1530/EJE-21-1109 (PMC9513639; doi:10.1530/EJE-21-1109)
Supplement: Supplementary Table 3. Reason for initial presentation [file supplementary_table_3.pdf]

Health Status of Children and Young Persons with Congenital Adrenal Hyperplasia in the UK (CAH-UK)

Supplementary Table 3. Reason for initial presentation

Presentation	Number (Percentage)
**Ambiguous genitalia***	35 (34.7%)
**Salt losing crisis***	26 (25.7%)
**Early puberty**	18 (17.8%)
**Premature pubarche, Tall stature**	7 (6.9%)
**Family screening, Prenatal diagnosis****	10 (9.9%)
**Poor feeding, Poor weight**	4 (3.9%)
**Other – investigation in the neonatal unit while admitted for prematurity**	1 (0.9%)

*Seven patients presented with both with atypical genitalia and salt losing crisis
**Only in one case was the mother treated with Dexamethasone prenatally
