# Supplementary material for: Health status of children and young persons with congenital adrenal hyperplasia in the UK (CAH-UK): a cross-sectional multi-centre study
Source: Eur J Endocrinol. 2022 Aug 24;187(4):543–53. doi: 10.1530/EJE-21-1109 (PMC9513639; doi:10.1530/EJE-21-1109)
Supplement: Supplementary Table 4. Medical background [file supplementary_table_4.pdf]

Health Status of Children and Young Persons with Congenital Adrenal Hyperplasia in the UK (CAH-UK)

Supplementary Table 4. Medical background

**Past medical history, Chronic conditions (number of patients) ***
**No other significant medical history (85)**
**Eczema / Atopy (4)**
**ASD / ADHD (4)**
**Anxiety and/or depression (3)**
**Precocious Puberty (3)**
**Type 2 Diabetes Mellitus (1)**
**Ulcerative colitis (1)**
**Renal dysplasia (1)**
**Other unrelated/minor conditions listed (16)**
Additional hormonal medication:<br>*GnRH antagonist (8 patients: 7 males, 1 female)*<br>*Oral contraceptives (3)*<br>*Desmopressin (1)*

*Study subjects listed multiple conditions
