# Supplementary material for: Health status of children and young persons with congenital adrenal hyperplasia in the UK (CAH-UK): a cross-sectional multi-centre study
Source: Eur J Endocrinol. 2022 Aug 24;187(4):543–53. doi: 10.1530/EJE-21-1109 (PMC9513639; doi:10.1530/EJE-21-1109)
Supplement: Supplementary Table 5. The results of biochemical investigations in patients with CAH in relation to the normal ranges of the respective local laboratories. [file supplementary_table_5.pdf]

Health Status of Children and Young Persons with Congenital Adrenal Hyperplasia in the UK (CAH-UK)

Supplementary Table 5. The results of biochemical investigations in patients with CAH in relation to the normal ranges of the respective local laboratories.

Test	Normal	High	Low	Result not available
**Sodium**	85.0%			15.0%
**Potassium**	83.0%		1%	16.0%
**Urea**	83.0%	2%		15.0%
**Creatinine**	75.0%	8.0%	2%	15.0%
**Plasma renin**	17.0%	31.0%	4.0%	48%
**Total cholesterol**	72.0%	3.0%	4.0%	21.0%
**HDL**	70.0%		3.0%	27.0%
**LDL**	60.0%	7.0%		33.0%
**Triglycerides**	75.0%	5.0%	1%	19.0%
**Glucose**	80.0%			20.0%
