# Supplementary material for: Health status of children and young persons with congenital adrenal hyperplasia in the UK (CAH-UK): a cross-sectional multi-centre study
Source: Eur J Endocrinol. 2022 Aug 24;187(4):543–53. doi: 10.1530/EJE-21-1109 (PMC9513639; doi:10.1530/EJE-21-1109)

Health Status of Children and Young Persons with Congenital Adrenal Hyperplasia in the UK (CAH-UK)

Supplementary Figure 2. Frequency of episodes of adrenal crisis requiring admission (n=35)

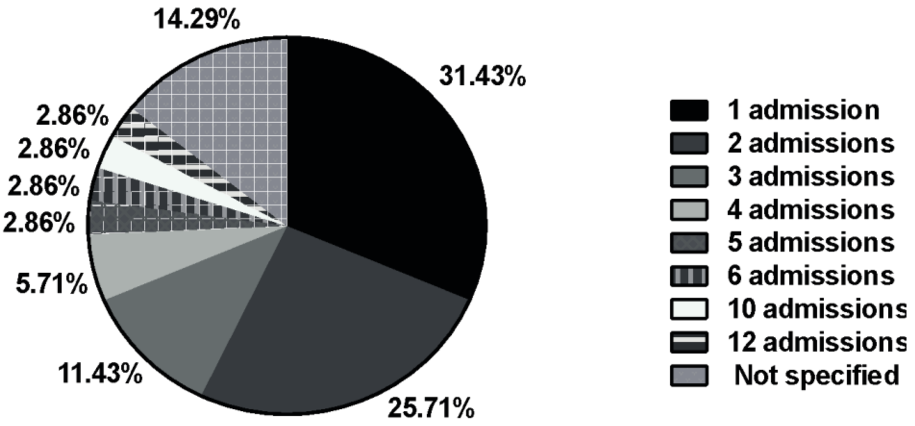

Supplement: Supplementary Figure 2. Frequency of episodes of adrenal crisis requiring admission (n=35) [file supplementary_figure_2.pdf]
