# Supplementary material for: Health status of children and young persons with congenital adrenal hyperplasia in the UK (CAH-UK): a cross-sectional multi-centre study
Source: Eur J Endocrinol. 2022 Aug 24;187(4):543–53. doi: 10.1530/EJE-21-1109 (PMC9513639; doi:10.1530/EJE-21-1109)

Health Status of Children and Young Persons with Congenital Adrenal Hyperplasia in the UK (CAH-UK)

Supplementary Figure 3 Daily absolute mineralocorticoid doses for sex (A) and age (B) groups, expressed as fludrocortisone µg/day. The horizontal lines correspond to the median, 25th and 75th quartiles. (*indicates statistical significance)

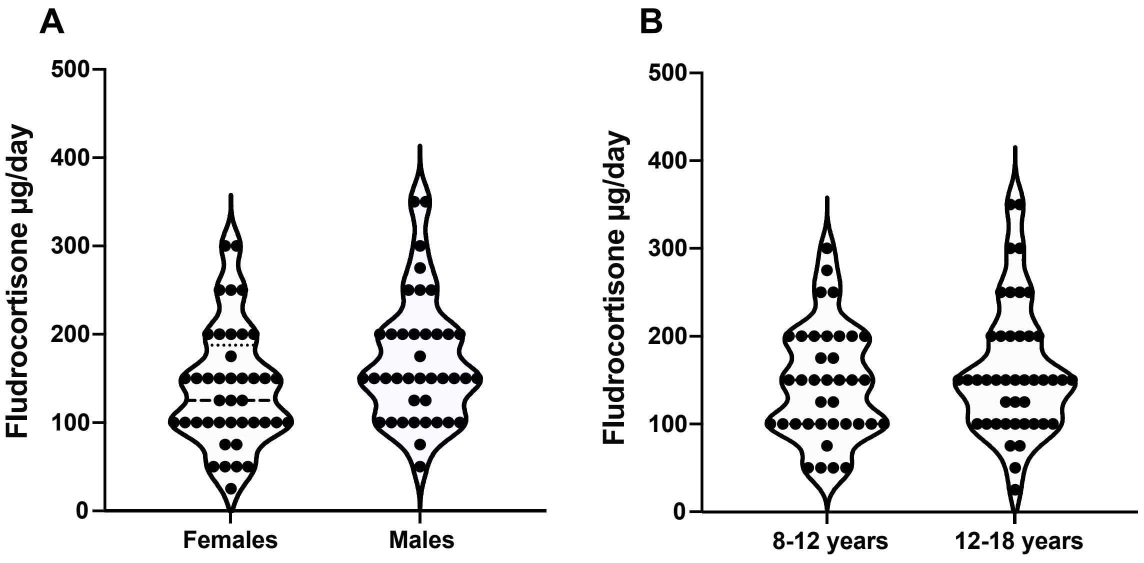

Supplement: Supplementary Figure 3 Daily absolute mineralocorticoid doses for sex (A) and age (B) groups, expressed as fludrocortisone µg/day. The horizontal lines correspond to the median, 25th and 75th quartiles. (*indicates statistical significance) [file supplementary_figure_3.pdf]
