# Supplementary material for: Health status of children and young persons with congenital adrenal hyperplasia in the UK (CAH-UK): a cross-sectional multi-centre study
Source: Eur J Endocrinol. 2022 Aug 24;187(4):543–53. doi: 10.1530/EJE-21-1109 (PMC9513639; doi:10.1530/EJE-21-1109)

Health Status of Children and Young Persons with Congenital Adrenal Hyperplasia in the UK (CAH-UK)

Supplementary Figure 5. Comparison of weight (A), height (B) and BMI (C) standard deviation scores (SDS) between patient subgroups of relative GC daily dose. Patients were divided between those receiving < 10, 10-15 and > 15 mg HC-equivalent/m^2/day. (D) Comparison of relative daily GC doses between patients with normal BMI and patients with BMI > 2SD, classified as overweight or obese. (*indicates statistical significance)

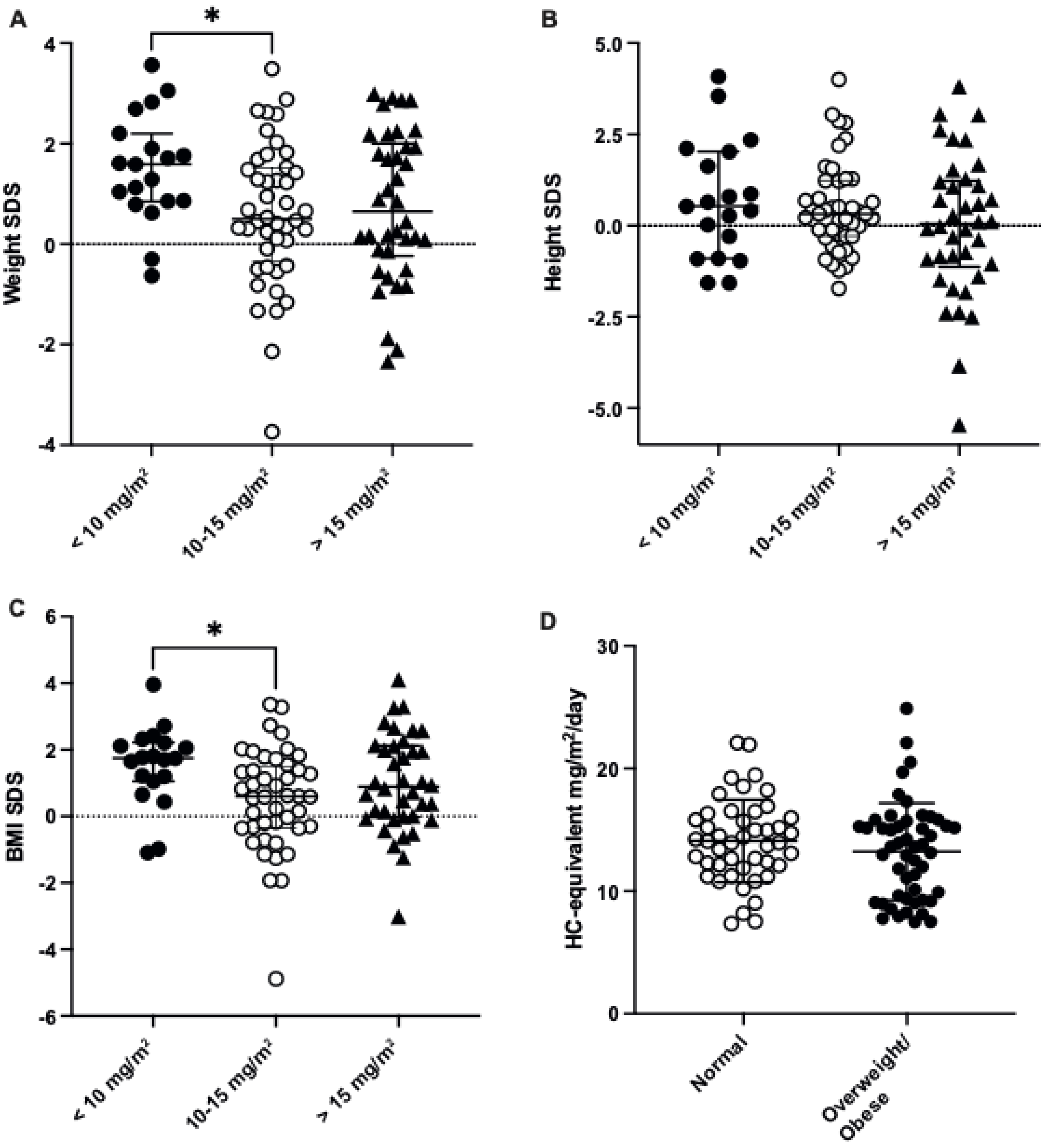

Supplement: Supplementary Figure 5. Comparison of weight (A), height (B) and BMI (C) standard deviation scores (SDS) between patient subgroups of relative GC daily dose. Patients were divided between those receiving < 10, 10-15 and > 15 mg HC-equivalent/m2/day. (D) Comparison of relative daily GC doses between  [file supplementary_figure_5.pdf]
