# Supplementary material for: Health status of children and young persons with congenital adrenal hyperplasia in the UK (CAH-UK): a cross-sectional multi-centre study
Source: Eur J Endocrinol. 2022 Aug 24;187(4):543–53. doi: 10.1530/EJE-21-1109 (PMC9513639; doi:10.1530/EJE-21-1109)

Health Status of Children and Young Persons with Congenital Adrenal Hyperplasia in the UK (CAH-UK)

Supplementary Figure 6. Paediatric Quality of life total scores in patients and parents (violin plots) compared to normative data, courtesy of Varni, et al. 2007. (boxes with error bars indicate mean with standard deviation)

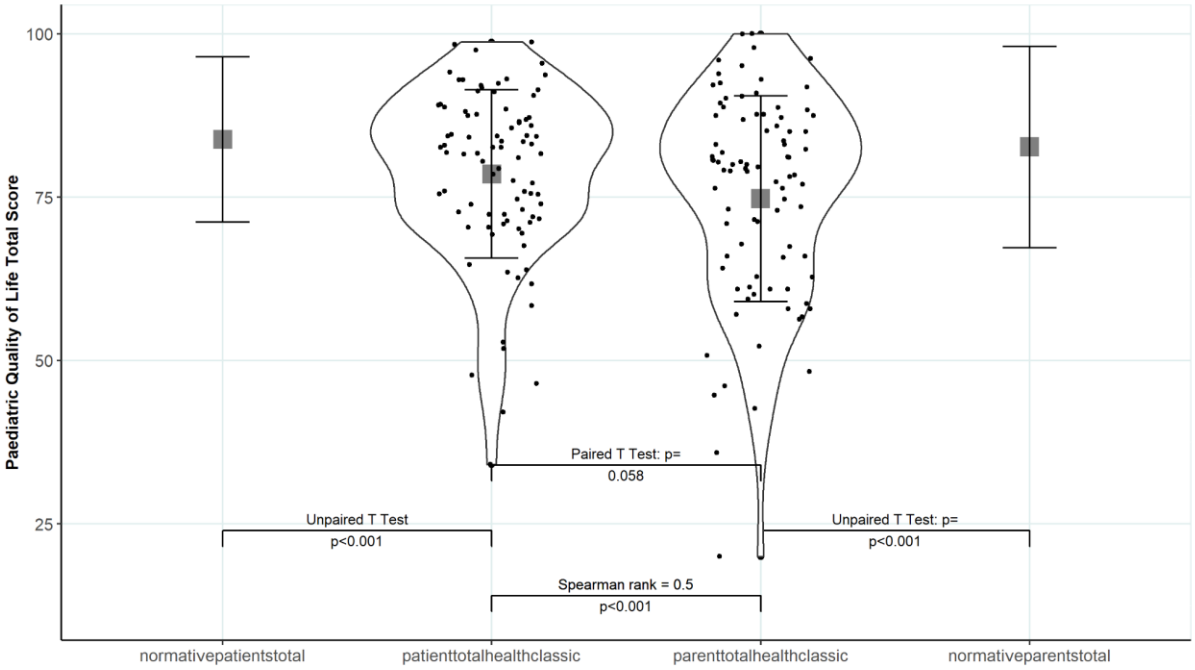

Supplement: Supplementary Figure 6. Paediatric Quality of life total scores in patients and parents (violin plots) compared to normative data, courtesy of Varni, et al. 2007. (boxes with error bars indicate mean with standard deviation) [file supplementary_figure_6.pdf]
